# Factors associated with health-related quality of life in women using path analyses: mediation effect of the adiposity traits

**DOI:** 10.1186/s12905-021-01535-7

**Published:** 2021-11-24

**Authors:** Mahdieh Khodarahmi, Mahdieh Abbasalizad Farhangi, Sahar Khoshro, Parvin Dehghan

**Affiliations:** 1 Department of Community Nutrition, Faculty of Nutrition, Tabriz University of Medical Sciences, Attar Neyshabouri Street, Tabriz, Iran; 2 Student Research Committee, Tabriz University of Medical Sciences, Tabriz, Iran; 3 Department of Nutrition and Biochemistry, Faculty of Nutrition, Tabriz University of Medical Sciences, Tabriz, Iran

**Keywords:** Body mass index, Sleep quality, Eating behavior, Quality of life

## Abstract

**Background:**

The current work aimed to investigate the mediating role of adiposity traits in the relationship between eating behaviors, sleep quality, socio-demographic factors, and the health-related quality of life in women of reproductive age in northwest of Iran.

**Methods:**

In the current cross-sectional study, a total of 278 overweight and obese women of reproductive age (20–49 y) were enrolled. Anthropometric assessments were performed. Pittsburgh sleep quality index (PSQI) was used for assessment of sleep quality while Short Form 36 (SF-36) questionnaire was used to measure health-related quality of life (HRQoL). Three-Factor Eating Questionnaire-R18 (TFEQ-R18) was used to measure eating behaviors. Path analysis was used to test the relationships between parameters.

**Results:**

Age was found to be indirectly and negatively associated with mental component score (MCS) (B = − 0.040; *P* = 0.049) and physical component score (PCS) (B = − 0.065; *P* = 0.036) through mediatory effects of obesity. Additionally, education was seen to be indirectly and positively related to MCS (B = 0.529; *P* = 0.045) and PCS (B = 0.870; *P* = 0.019), respectively. On the other hand, obesity (B = 0.608; *P* = 0.018) and PSQI score (B = − 0.240; *P* = 0.034) had direct associations with MCS. Age (B = − 0.065; *P* = 0.036) and education (B = 0.870; *P* = 0.019) were also directly associated with obesity.

**Conclusions:**

Obesity seemed to mediate the effects of socio-demographic parameters on HRQoL. Poor sleep quality was also related to impairment of HRQoL. Further studies are needed to confirm these results.

## Background

Obesity as one of the most vital challenges around the world has been associated with social and medical complications and is known as the epidemic of the twenty-first Century [1]. The prevalence of obesity has increased in both developed and developing countries [2]. In many countries, obesity is the leading modifiable risk factor for a number of diseases including coronary artery disease, type 2 diabetes mellitus, hypertension, hyperlipidemia, some types of cancer, sleep apnea, stroke, liver diseases, gynecological problems, osteoarthritis, and other adverse pathological conditions [3]. Obesity is characterized by the excessive fat accumulation in the body and results from an imbalance between energy intake and expenditure [4]. The etiology of obesity is multi-factorial including a combination of environmental, biological, emotional, genetic, social, and behavioral determinants [5, 6]. Obesity is also an important health problem in Iran. The most important reason of recorded mortalities in 2002 in Iran was obesity and overweight [7]. The prevalence of obesity was 24% for women and 18% for men in the north-west of Iran in 2006 [8]. Men and women are different in total body fat distribution and women have a higher percentage of body fat and less lean mass and accordingly higher prevalence of obesity than men [9]. Obesity in women in reproductive age is a risk factor for the health of women and their children. It has been shown that obese women than normal women are at a high risk for reproductive health such as infertility, and also have higher risks associated with pregnancy and birth, lower self-esteem and higher depression [10–12].

Quality of Life is significantly affected by the clinical status and complications of obesity. The World Health Organization (WHO) defines the health-related quality of life (HRQoL) as an individuals’ perception of their position in life in the context of the culture and value systems in which they live and in relation to their expectations, goals, concerns and standards. HRQoL includes: mental, emotional, physical, and social aspects [13]. In fact, HRQoL has two basic principles: the first part, which contains psychological, social, physical and emotional fields. The second part is subjective and is reported based on personal experiences [14]. Many studies have focused on the effects of obesity on the HRQoL and the previous studies have shown a close negative association between the HRQoL and obesity [15]. Several studies have shown that obese people have lower scores of HRQoL than normal-weight subjects and increased weight was associated with lower role physical, physical functioning, bodily pain, vitality, and general health scores [15–17]. In general, obesity reduces HRQoL and treatment of obesity improves HRQoL [18].

Several studies have shown that obesity is associated with common mental disorders such as anxiety, depression, low self-esteem, and lower quality of life as well, and on the other hand, weight loss can improve psychological well-being [19–24]. Additionally, researchers have suggested that psychological factors may alter eating behaviors and food choices and lead to increased intake of high energy dense foods which predispose subjects to obesity and its-related complications [25]. Eating disorders are common especially among young women and adolescent girls [26]. In addition, previous studies have shown that inappropriate eating behaviors can predict weight gain and obesity in women [27–29]. For instance, cognitive dietary restraint and disinhibited eating in overweight and obese individuals than underweight and normal weight have reported to be higher [30, 31]. Moreover, it has been revealed women have higher scores of these factors compared with men [32, 33]. Keranen et al. reported that high cognitive restraint, with low emotional eating and uncontrolled eating was associated with maintained weight loss [34]. High score in the cognitive restraint has been associated with a reduction in energy and food intake while the loss of control over eating (high disinhibition) can cause excessive energy intake and increased body weight [27, 35].

Sleep is one of the most important human behaviors that occupies between 20 and 40% of the day [36]. Investigations have revealed that sleep disruptions are highly prevalent in middle-aged women [37]. On the other hand, neurologic effects of sleep deprivation can lead to increased caloric intake and obesity and there are several studies that have shown these associations [38, 39]. Sleep loss and sleep disorders due to lifestyle changes are common problems in the modern world. There are some studies that have shown sleep deprivation may result in detrimental effects on the body's endocrine system and its metabolism [40] and subsequently, increased risk of obesity and overweight [41], cardiovascular disease [42], and diabetes [43–45].

Taken together, obesity and its-related health outcomes can be influenced by a variety of factors (behavioral, environmental, lifestyle and genetic factors) which are closely inter-related. Thus, unmeasured interrelationships and high colinearity which exist between the variables under investigation limit the understanding of how all of these factors directly and indirectly may influence the association between above-mentioned factors and obesity and its-related health problems. On the other hand, usual statistical techniques such as traditional regression models which have been used in most studies focus only on the association between a limited number of predictors and a single outcome [46]. Path analysis is an extension of multiple regression that allows researchers to assess direct and indirect effects by considering multiple independent and dependent variables simultaneously [47]. Path analysis represents fit of a correlation matrix with a causal model that researchers want to test. In fact, a proper path analysis can provide more profound understanding of the interrelations of the variables [47]. There is no study that has applied the path analysis and simultaneously examined the role of all these parameters in development of obesity and its consequences. So, the aim of our study was to apply path analysis in overweight and obese women to determine the direct and indirect associations of socio-demographic, eating behaviors, sleep quality and adiposity traits with health-related quality of life.

## Methods

### Participants

**Fig. 1:**
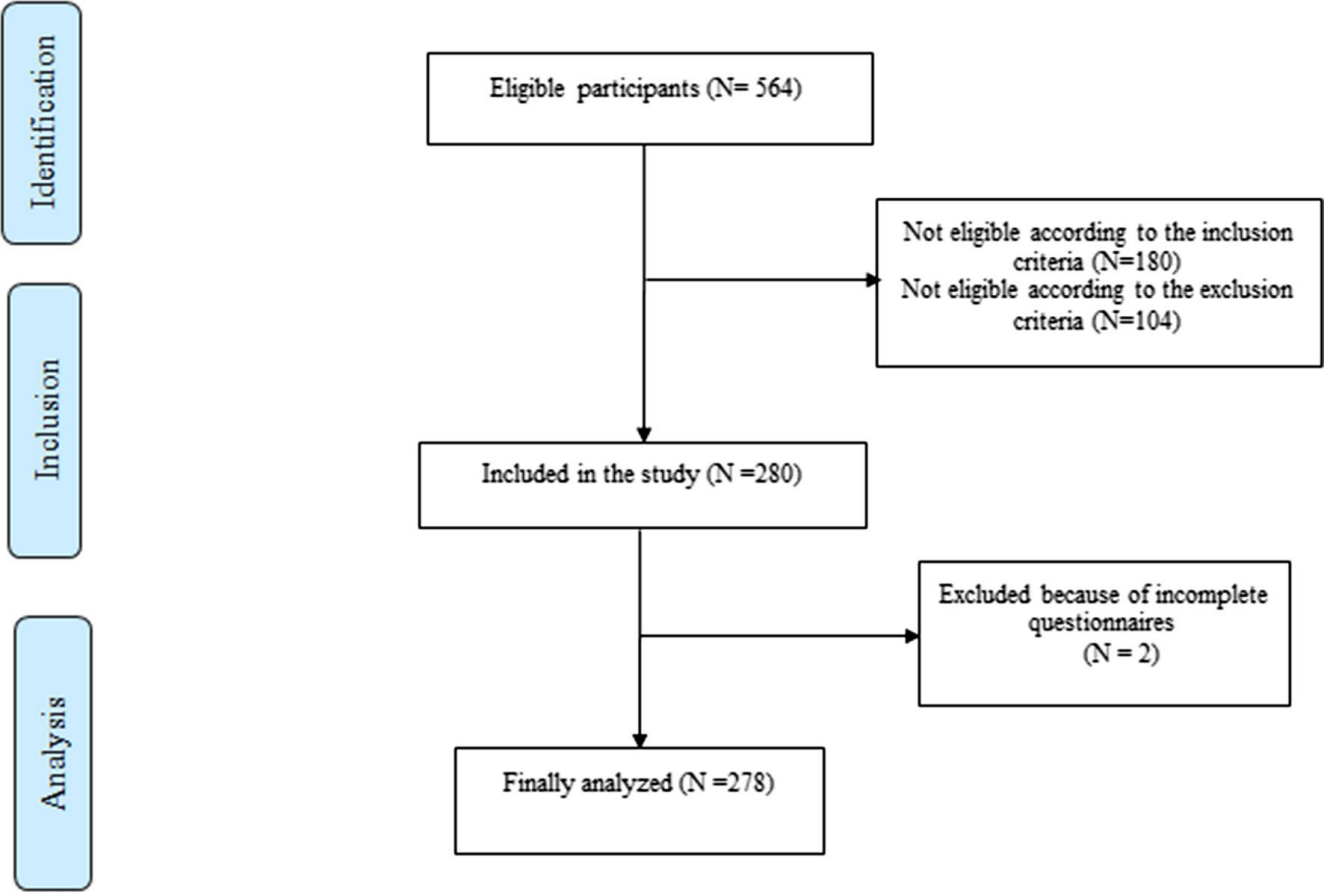
Strobe flow-diagram of study participants

A total of 278 women in reproductive age (20–49 years) were enrolled in the current cross-sectional study from November 2017 to December 2018. The eligible participants were recruited using convenience sampling through announcements and posters placed in public areas of the city (Tabriz). Inclusion criteria targeted apparently healthy women (without any specific disease that affects diet or appetite) who had BMI > 25 kg/m^2^. At first, a total of 564 women applied to take part. Of these, after application of the inclusion and exclusion criteria, 284 women were excluded. Finally, 278 adult female subjects met the inclusion criteria and were recruited in the study. A completed flow-diagram of the study is presented in Fig. 1. All of participants completed written informed consent prior to participation in this study. With maximum RMSEA of 0.1 [48], α = 0.05 and power of 80%, a minimum sample size (n) = 184 was calculated using statistica software, version 10. Totally, a sample of 278 participants who agreed to take part was evaluated in the current study. The protocol of the study was approved by the ethical committee of Tabriz University of Medical Sciences (IR.TBZMED.REC.1400.294). All of information was collected through face to face interview by trained dietitians.

### Anthropometric measurements

Anthropometric variables including height, weight, waist and hip circumference (WC and HC) were measured by an expert nutritionist. Height was measured by a stadiometer fixed to the wall with a precision of 0.5 cm, while subjects were in light clothing without shoes. Body weight was measured by a digital scale while participants were minimally clothed without shoes. WC was measured by a tape measure to the nearest 0.5 cm, at the midpoint between the last costal rib and the iliac crest without applying any pressure to the body. HC was also measured at the largest part of the hip. BMI was calculated as body weight (kg) divided by the square of height (m^2^). Obesity was defined as having BMI ≥ 30 kg/m^2^.

### Sleep quality assessment

Information on sleep quality was collected using a self-administered Pittsburgh Sleep Quality Index (PSQI) questionnaire [49]. Validity and reliability of the questionnaire have been previously confirmed in Iran [50]. This scale was a 19-item self-report questionnaire which includes seven sub-scales containing: sleep latency, sleep quality, habitual sleep efficiency, sleep duration, daytime dysfunction, use of sleeping medications and sleep disturbances [49]. Each component was rated from 0 to 3 and global score computed by summing the seven sub-scales. The total score ranged from 0 to 21 points and the higher scores indicated poor sleep quality.

### Health-related quality of life (HRQoL) assessment

HRQoL was evaluated by a short form 36 (SF-36) questionnaire, with prior evidence of validity and reliability among Iranian population [51]. This self-administered questionnaire which consists of 36 items calculates eight subscales: physical functioning (10 items), role physical (four items), bodily pain (two items) and general health perception (five items), role emotional (three items), vitality (four items), mental health (five items) and social functioning (two items) [52, 53]. Scores of the first four dimensions were summed to create the physical component score (PCS), whereas the last four were summed to form the mental component score (MCS). Scores for each dimension ranged from 0 to 100, high scores showing a better health status in people [53]. Subjects whose scores were less than 66.7 percent were considered poor HRQoL.

### Three-Factor Eating Questionnaire-R18 (TFEQ-R18)

TFEQ-R18 is a common instrument to study eating behavioral pattern in obese people, as well as for predicting weight loss in clinical patients and monitoring progress throughout treatment [54, 55]. TFEQ-R18 is a shortened and revised version of the original 51-item TFEQ [56]. TFEQ-R18 measures 3 different aspects of eating behavior: uncontrolled eating, emotional eating and cognitive restraint. Cognitive restraint is defined as conscious restriction of food intake to control body weight (six items). Emotional eating refers to the inability to resist emotional cues (three items); while uncontrolled eating is defined as tendency to eat more than usual due to a loss of control over intake accompanied by subjective feelings of hunger (nine items) [57]. Subjects were requested to respond to 18 questions on a 4-point response scale (1–4). Then, items scores were summated into subscale scores and the higher scores in the scales were indicative of greater uncontrolled, cognitive restraint, or emotional eating. Validity and reliability of this questionnaire were previously confirmed for using in Iran. The Cronbach's alpha coefficient for this questionnaire in Iranian subjects was 0.699 which was very close to the acceptable level [58].

### Statistical analyses

Normal distribution of data was checked by descriptive measures such as coefficients of skewness and kurtosis, mean and standard deviation. Data on quantitative and qualitative characteristics were expressed as the mean ± SD and frequency (%), respectively. Path analysis was run to comprehensively investigate the associations of socio-demographic, sleep quality, eating behaviors and health-related quality of life which has been hypothesized to be associated with obesity. In fact, path analysis is an extension of multiple regressions which allows simultaneous analysis of relationships between dependent variables as well as between independent variables and dependent variables. Maximum Likelihood Estimation Procedure (ML) was used to estimate regression coefficients. The model fitness was evaluated using multiple fit indices including: comparative fit index (CFI) > 0.90 [58], Tucker-Lewis index (TLI) ≥ 0.90 [59], chi-square test (χ^2^/degrees of freedom (df) ratio < 5 [59], standardized root mean square residual (SRMR) < 0.08 [48] and root mean square error of approximation (RMSEA) ≤ 0.08 [48]. The statistical analyses were performed using STATA V.15.0. *P*-values less than 0.05 were considered statistically significant.

## Results

A summary of the demographic characteristics of study participants is shown in Table 1. The mean age and BMI of subjects were 31.40 ± 10.89 years and 29.19 ± 4.14 kg/m^2^, respectively. In terms of marital status, 51.4% of women were married, 2.9% were widows, 2.9% were divorced and 42.8% were single. Moreover, the majority (72.3%) of participants were unemployed and about 27.7% of were employed. Table 2 provides the descriptive statistics for the Three-Factor Eating Questionaire-R18 scores, sleep quality, and physical and mental component score of the Short Form-36. In the overall population, the scores of the two main domains, PCS and MCS, were 75.0% and 71.6%, respectively. Thus, majority of participants had better quality of life.

**Table 1:** Descriptive characteristics of the study subjects (N = 278)

Variables	Mean or N	SD or %
Age (Year)	31.40	10.89
Weight	76.31	10.52
BMI (kg/m^2^)	29.19	4.14
WC (cm)	95.23	12.06
WHR	0.85	0.08
**Marital status**		
Married	143	51.43
Widowed	8	2.88
Divorced	8	2.88
Single	119	42.81
**Education**		
Illiterate	8	2.88
Elementary.8 years	39	14.03
Intermediary.12 years	63	22.66
More12 years	168	60.43
**Occupational status**		
Employed	77	27.70
Unemployed	201	72.30
**Household size**		
≥ 4	202	72.66
< 4	76	27.34

BMI, body mass index; WC, waist circumference; WHR, waist-to-hip ratio

**Table 2:** Three-Factor Eating Questionaire-R18 scores, sleep quality, physical and mental component score of the Short Form-36 (n = 278)

Sleep quality and dimensions of health related quality of life and eating behavior	Mean (SD)	Minimum	Maximum
TFE cognitive score	15.72 (3.57)	6.00	24.00
TFE uncontrolled score	20.03 (4.91)	9.00	32.00
TFE emotional score	6.58 (2.66)	3.00	12.00
PSQI score	6.38 (3.73)	0.00	16.00
PCS score	75.00 (17.30)	0.00	100.00
MCS score	71.66 (17.49)	0.00	100.00

TFE, Three-Factor Eating; PSQI, Pittsburgh Sleep Quality Index; PCS, physical component score; MCS, mental component score

**Fig. 2:**
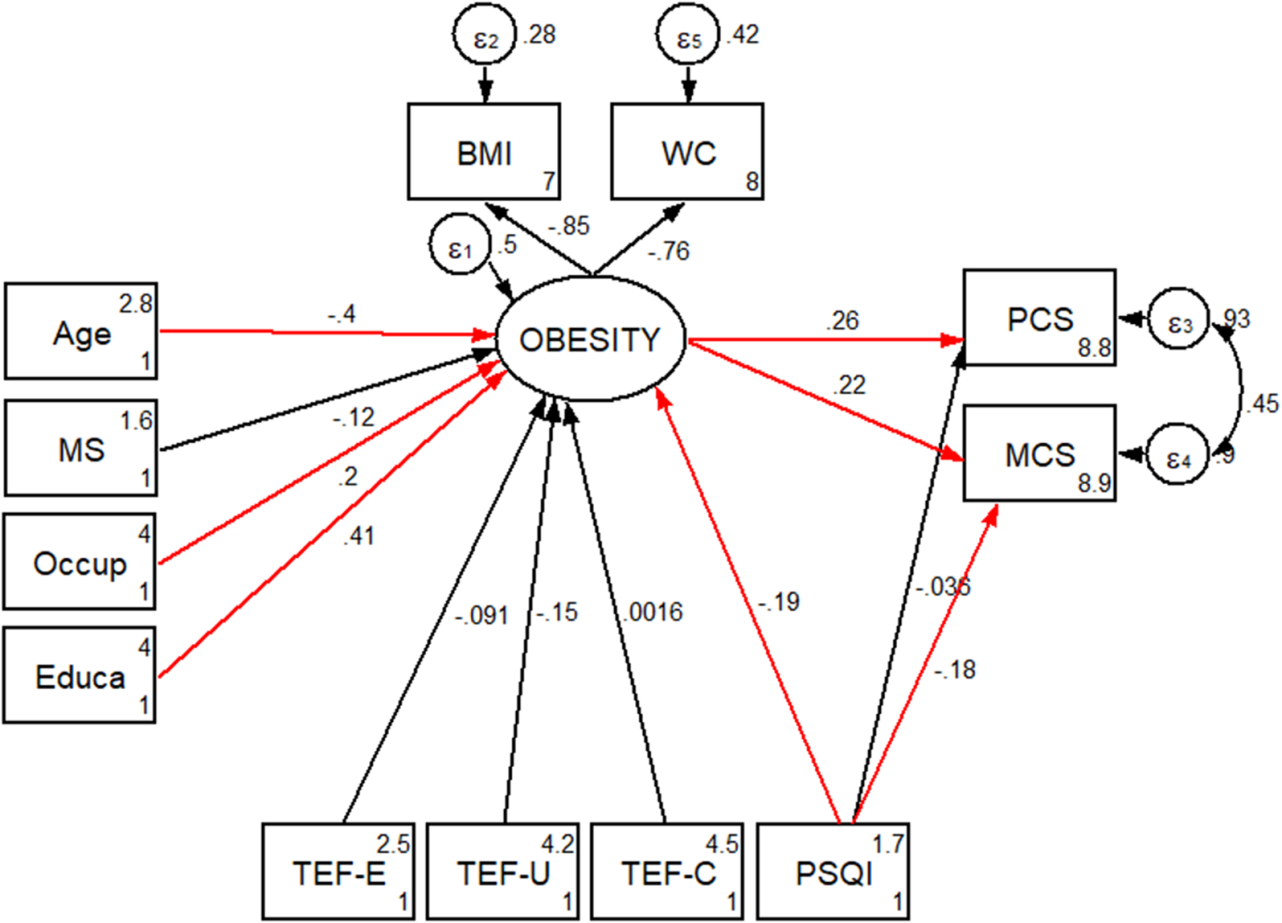
Structural equation model diagram with standardised estimates for total effects of socio-demographic, sleep quality, eating behaviors, and obesity on health related quality of life among women of reproductive age. Abbreviations: Occup, occupation; Educa, education; MS, marital status; BMI, body mass index; WC, waist circumference; MCS, mental component score; PCS, physical component score; PSQI, Pittsburgh sleep quality index; TFE-E, three factor eating-emotional eating; TFE-U, three factor eating-uncontrolled eating; TFE-C, three factor eating-cognitive restraint. *All path coefficients are standardized. Red arrows mean *p*.value ≤ 0.05. ^£^Total effect is defined as the sum of direct and indirect effects

The mean of PSQI global score was 6.38 which indicating poor sleep quality (PSQI total score of > 5 is indicative of impaired sleep quality). Mean scores for the TFEQ-R18 factors were 15.72 for cognitive restraint, 20.03 for uncontrolled and 6.58 for emotional. These higher scores of these scales denote higher levels of uncontrolled, cognitive restraint, or emotional eating. Significant direct and indirect paths of the association between socio-demographic, sleep quality, eating behaviors, health-related quality of life and obesity among women are presented in Table 3. Among socio-demographic parameters, age was found to be indirectly and negatively associated with MCS (B = − 0.040; *P* = 0.049) and PCS (B = − 0.065; *P* = 0.036) through mediatory effects of obesity. Additionally, education was seen to be indirectly and positively related to MCS (B = 0.529; *P* = 0.045) and PCS (B = 0.870; *P* = 0.019), respectively. On the other hand, obesity (B = 0.608; *P* = 0.018) and PSQI score (B = − 0.240; *P* = 0.034) had positive and negative direct associations with MCS, respectively. Age (B = − 0.065; *P* = 0.036) and education (B = 0.870; *P* = 0.019) were also directly associated with obesity. Presented model provided a good fit (χ^2^/df = 1.59; CFI = 0.931; TLI = 900; RMSEA (95% CI) = 0.066 (0.018–0.104); SRMR = 0.040). Path analysis diagram with standardized estimates illustrating the total effects of socio-demographic, sleep quality, eating behaviors, and obesity on health-related quality of life is shown in Fig. 2.

**Table 3:** Statistically significant direct and indirect pathways of the association between socio-demographic, sleep quality, eating behaviors, health related quality of life and obesity among women using path analysis

Model path	Standardized estimate	SE	P	95% CI
Direct effects				
Obesity → MCS	0.608	0.258	0.018	(0.10, 1.11)
PSQI → MCS	− 0.240	0.113	0.034	(− 0.46, − 0.02)
Age → Obesity	− 0.065	0.031	0.036	(− 0.13, − 0.01)
Education → Obesity	0.870	0.370	0.019	(0.15, 1.59)
Indirect effects				
Age → MCS	− 0.040	0.021	0.049	(− 0.08, − 0.01)
Education → MCS	0.529	0.264	0.045	(0.01, 1.05)
Age → PCS	− 0.065	0.031	0.036	(− 0.13, − 0.01)
Education → PCS	0.870	0.370	0.019	(0.15, 1.59)

CI, confidence interval; MCS, mental component score; PSQI, Pittsburgh sleep quality index; PCS, physical component score; SE; standard error of the estimate

All standardized path coefficients shown were significant (*P* < 0.05)

## Discussion

The aim of the present study was to investigate the mediating role of adiposity traits in the relationship between eating behaviors, sleep quality, socio-demographic factors, and the health-related quality of life by path analysis in women of reproductive age. In the current work, we found that obesity mediates the association of age and education with health-related quality of life (MCS and PCS). On the other hand, a positive direct association between obesity and MCS was found. Higher PSQI score was directly and negatively related to MCS. Unexpectedly, a direct positive relationship between education and obesity among women was also identified.

Our finding regarding an indirect negative association between age and physical and psychological dimensions of HRQoL suggested that younger people had better health condition than older people. Interestingly, we found a direct positive association between obesity and MCS which it could be concluded that mental aspect of health-related quality of life was favorably influenced by overweight (mean of BMI = 29.1). It should be taken into account that in Iran similar to other west Asian countries which mostly are Muslim, covering of body particularly in women is a law which can result in better body satisfaction [60]. In this regard, there are some reports among women that have shown being overweight but not obese is perceived ideal and desirable body image [61]. On the other hand, in some Asian cultures, fatness is still considered as a symbol of wealth while leanness is perceived as poor [62]. Interestingly, it seemed that being overweight in the current population that were young and in reproductive age (mean age = 31 years), had a protective role to decrease mental aspect of health-related quality of life. Thus, since in the present study, age was inversely related to the obesity, it is not surprising that being overweight could indirectly mediate the negative association of age with mental health-related quality of life. However, no significant association was found between age and physical health domain of SF 36. Previous studies have shown that obesity is related to worse HRQoL among different populations, particularly in the physical dimensions and among female subjects [63]. These studies have also revealed that higher degrees of obesity are related to greater impairment of HRQoL which might be due to increased joint pain and decreased physical activity [63]. For example, the results of 2 large prospective cohorts of US women indicated that weight gain during a 4-year period was associated with worse HRQoL in the dimensions of physical functioning, role limitations due to physical problems, bodily pain, general health, and vitality [64]. Another cohort study by Molero and et al. which examined impact of obesity on HRQoL using the SF-36 reported an adverse effect of obesity on HRQoL, influencing the physical dimension more significantly than the psychosocial dimension [65]. On the other hand, it has been shown that lower HRQoL is significantly related to higher age [66]. Nevertheless, other studies have proposed a U-shaped relationship across the course of life, with a drop in quality of life and wellbeing in oldest age groups [67].

Noticeably, we found the positive indirect associations of education with physical and mental subscales of HRQoL. Additionally, a direct positive relationship between education and obesity was also observed in the current research. In agreement with our results, some studies in low-income countries have shown that a higher education seems to be related to a higher likelihood of being obese [68]. However, it appeared that the association of educational attainment with obesity might rely on the level of development in the country, such that negative relationships were more common in developed countries [69]. In line with our findings, a systematic review reported that participants with higher levels of education in developing countries were more likely to be obese [68]. Lifestyle factors which are influenced by education and other socio-economic parameters may be the keys for understanding the education-obesity relationship [70]. Another main finding in the present research was the direct negative association between PSQI score and MCS. In other words, poor sleep quality was related to impairment of health-related quality of life. A wealth of evidence has shown that insufficient and poor-quality sleep may contribute to increasing health burden. The result of current study pertaining to the associations between sleep quality and MCS study are in accordance with previous studies that have reported sleep disorders are associated with impairment of HRQoL [71]. Additionally, in our study, subjects who were overweight or obese had a higher global PSQI score which was in line with previous findings that the PSQI score was correlated with obesity in female African Americans. It seems that sleep deprivation and sleep disorders may through alterations in hormonal status such as increase in ghrelin level and decrease in leptin level lead to obesity, metabolic dysregulation and obesity-related complications [72].

Several limitations of the current study should also be addressed; first of all, the cross-sectional design of the current study makes impossible causal inferences and large longitudinal studies are needed to infer true causal associations. Moreover, dietary factors might influence studied parameters and it would be better to study their associations with desired variables. Since dietary behaviors and other lifestyle factors in Tabriz may vary from those in other parts of the country, and on the other hand, due to using convenience sampling instead of random sampling method in this research, our results cannot be generalized to all Iranian population. Nevertheless, this study is the first to evaluate the association between eating behaviors, sleep quality, socio-demographic factors, adiposity traits and the health-related quality of life simultaneously by path analysis which provides important information for decision makers on health issues and may be useful for effective primary-prevention strategies for obesity.

## Conclusion

In summary, the findings of the present study suggested an indirect association between some of the socio-economic factors (age and education) and health related quality of life (MCS and PCS). Additionally, a direct relationship between obesity and outcome parameter (MCS) was revealed. Poor sleep quality was also related to impairment of health-related quality of life. Prospective studies are warranted to clarify and confirm the possible associations of this kind.

## Data Availability

The datasets used and/or analyzed during the current study available from the corresponding author on reasonable request.

## Abbreviations

BMI: Body mass index
HRQoL: Health related quality of life
SF-36: Medical Outcomes Study Short Form 36
MCS: Mental component score
PCS: Physical component score
